# An Enriched Rearing Environment Calms Adult Male Rat Sexual Activity: Implication for Distinct Serotonergic and Hormonal Responses to Females

**DOI:** 10.1371/journal.pone.0087911

**Published:** 2014-02-05

**Authors:** Susumu Urakawa, Dai Mitsushima, Michito Shimozuru, Yasuo Sakuma, Yasuhiko Kondo

**Affiliations:** 1 Department of Physiology, Nippon Medical School, Tokyo, Japan; 2 Department of Judo Neurophysiotherapy, Graduate School of Medicine and Pharmaceutical Sciences, University of Toyama, Toyama, Japan; 3 Department of Systems Neuroscience, Graduate School of Medicine, Yamaguchi University, Yamaguchi, Japan; 4 Department of Environmental Veterinary Sciences, Hokkaido University, Hokkaido, Japan; 5 Laboratory of Veterinary Ethology, The University of Tokyo, Tokyo, Japan; 6 Department of Animal Sciences, Teikyo University of Science, Tokyo, Japan; CNRS, France

## Abstract

Early life events induce alterations in neural function in adulthood. Although rearing in an enriched environment (EE) has a great impact on behavioral development, the effects of enriched rearing on sociosexual behavior remain unclear. In this study, we investigated the effects of rearing in an EE on male copulatory behavior and its underlying neurobiological mechanisms in Wistar-Imamichi rats. Three-week-old, recently weaned rats were continuously subjected to a standard environment (SE) or an EE comprised of a large cage with several objects, such as toys, tunnels, ladders, and a running wheel. After 6 weeks, rats reared in an EE (EE rats) showed decreased sexual activity compared with rats reared in a SE (SE rats). This included a lower number of ejaculations and longer latencies in three consecutive copulatory tests. In addition, EE rats showed decreased emotional responsiveness and less locomotor behavior in an open field. In a runway test, on the other hand, sexual motivation toward receptive females in EE males was comparable to that of SE males. Furthermore, following exposure to a female, increases in serotonin levels in the nucleus accumbens and the striatum were significantly suppressed in EE males, whereas dopaminergic responses were similar between the groups. Female-exposure-induced increases in the levels of plasma corticosterone and testosterone were also suppressed in EE rats compared to SE rats. These data suggest that rearing in an EE decreases male copulatory behavior, and serotonin and hormonal regulating systems may regulate the differences in sociosexual interactions that result from distinct rearing environments.

## Introduction

In rodents, rearing in enriched environment (EE)—such as in large cages containing toys, tunnels, ladders, running wheels, other enriching items, or several cage mates—is generally believed to facilitate enhanced motor, sensory, social, and cognitive functions compared with rearing in a standard environment (SE, laboratory cages) [1], [2], [3]. A number of studies have shown significant effects of EE rearing on brain plasticity and subsequent adult behavior, with EE rearing facilitating recovery from brain dysfunctions following lesions [4], [5], enhancing learning and memory [6], [7], and increasing exploratory and decreasing anxiety-like behavior [8], [9], [10], [11]. In contrast, early-life stress may produce effects opposite to EE. Isolated rearing and maternal separation during the neonatal period increases anxiety-like behavior, play behavior, and intermale aggression [12], [13], [14], [15], [16], whereas prepubertal experience of an EE or social housing (several animals living together in a cage) decreases play fighting behavior and social interactions [13], [14], [17], [18].

Many factors could contribute to the modification of emotional or social behavior by rearing environment. Midbrain serotonin (5-HT) and dopamine (DA) neurotransmitter systems have been implicated in emotionality. 5-HT neurons located in the raphe nuclei project to many brain areas, including the limbic system, and have been shown to be involved in the modulation of anxiety (for reviews, see Refs [19], [20]). On the other hand, the mesocorticolimbic dopaminergic system has been mainly related to reward and motivation (for reviews, see Refs [21]). Furthermore, the hypothalamic-pituitary-adrenal (HPA) axis is an important modulator of stress-related behavior. HPA activity is reflected peripherally by plasma concentrations of corticosterone. In a study of the physiological effects of EE on stress responses, Beltz et al., reported that EE decreased corticosterone concentrations in isolated rats [22].

Although recent studies have led to a better understanding of the effects of rearing environment on emotional and social behavior via neuronal and hormonal regulator systems, the influence of EE rearing on sexual behavior, one of the most important social behaviors, remains unclear. In the present study, we investigated the consequences to male rats of being reared in an EE (EE rats) from early adolescence to puberty on adult sexual behavior, in comparison to rats reared in a SE (SE rats). Furthermore, to reveal the neurobiological mechanisms underlying the behavioral effects of EE rearing, we focused on the neurotransmitter 5-HT and DA and the hormone corticosterone and testosterone, which have been implicated in the neural regulation of sexual and emotional behavior [23], [24], [25], [26], [27]. In the present study, we examined the effects of EE rearing on: 1) sexual (copulatory) and emotional behavior, 2) serotonergic and dopaminergic activity following female exposure, and 3) corticosterone and testosterone responses following female exposure.

## Materials and Methods

### Animals and housing conditions

Male and female rats of the Wistar-Imamichi strain were obtained from the Institute for Animal Reproduction (Ibaraki, Japan). The rats were maintained under a controlled temperature (23±2°C) and a reversed illumination cycle (lights on from 23:00 to 11:00). Food and water were available ad libitum. All experimentation and animal housing adhered to the guidelines for the Care and Use of Laboratory Animals of Nippon Medical School, and were approved by the Laboratory Animals Ethics Committee of Nippon Medical School.

At postnatal day 25, male rats were housed in SE cages (40×23×18 cm, 2–3 rats in each laboratory cage, n = 14) or EE cages (81×51×53 cm, 7 rats per cage, n = 14) that were equipped with horizontal platforms and various toys such as a running wheel, tunnels, a climbing ladder, wooden blocks, and a bridge and maze as described previously [5], [11]. The spatial arrangement of the objects was changed, and some of the toys were replaced with new toys twice a week. Females (n = 28) were housed in SE cages. All behavioral tests described below were carried out during the dark phase of the light-dark cycle.

### Open-field test

At postnatal day 60, all experimental males were tested for open-field behavior. The test was started by placing each male in the center (30-cm diameter) of a circular open-field arena (60-cm diameter, surrounded by a 50-cm wall). Behavior was video recorded for 20 min using a digital video camera (DCR-TRV30, SONY, Tokyo, Japan) and male locomotive behavior was analyzed off-line using commercial software (TopScan ver 1.00, Clever Sys., Inc., VA, USA). The open field floor was composed of a green sheet of paper to provide contrast for the image analysis software. The sheet was changed between every each trial.

### Copulatory behavior test

All stimulus females (8 weeks old) were ovariectomized under anesthesia with pentobarbital (50 mg/kg, i.p.) 2 weeks prior to their first use in copulatory behavior tests. They were injected with estradiol benzoate (Sigma, MO, USA: 5 µg in 0.1 ml sesame oil) 48 h before the tests and progesterone (Wako, Osaka, Japan: 500 µg in 0.1 ml sesame oil) 4 h before the tests. Starting at 1 week after the open-field test, when they were approximately 10 weeks old, copulatory behavior was tested three times weekly with same male-female partner in the same circular open field. The floor of the open field was again covered with a green sheet of paper for contrast. A pair composed of an experimental male and a stimulus female was placed in the center area and video recorded for 15 min. Copulatory behavior was analyzed off-line with appropriate software (TopScan ver 1.00). This analysis determined the (1) intromission latency (the time from the start of the test to the first intromission), (2) ejaculation latency (the time from the first intromission to the first ejaculation), (3) postejaculatory interval (PEI; the time from the first ejaculation to the next intromission), (4) mount frequency (MF; the number of mounts during the test period), (5) intromission frequency (IF; the number of intromissions during the test period), (6) the number of ejaculations during the test period, (7) inter intromission interval until the first ejaculation (III), and (8) intromission ratio (IR; IF/[MF + IF]). The criteria for mounts, intromissions, and ejaculations were described previously [28].

### Runway test

One week after finishing the copulatory behavior test (when the animals were approximately 13 weeks old), sexual motivation toward a receptive female was measured using a straight runway procedure modified from [29]. The apparatus consisted of a start box (25×25×25 cm), an alley (150×12×12 cm) with a goal area (15×12×12 cm opposite to the start box), and a stimulus box (25×25×25 cm) connected to the goal area. A guillotine door was located between the start box and the alley, and the goal area and the stimulus box were divided by an aluminum board with holes that was screened to prevent direct contact between the stimulus females. The start and stimulus boxes had air windows (mesh-covered holes, 10 cm in diameter) on the opposite side of the alley. A motor-driven blower, attached to a window in the start box via a flexible duct, introduced air flux (approximately 0.2 m^3^/min) from the start to the goal (for habituation prior to the test) and from the goal to the start (during the test to convey the odor cues from the stimulus box). Before each test, the apparatus was cleaned with 70% ethanol (v/v).

Previously, all male subjects were allowed to individually explore the empty runway apparatus for 5–7 min on 2 separate days. This procedure was designed to habituate the males to the runway environment. On the test day, each male subject underwent four trials. This included an initial trial with no stimulus female (as baseline) and three successive trials with a receptive female (primed with sex steroids as described in the copulatory behavior test section). In each trial, a subject was placed into the start box that received air flux from outside the alley. After a 3-min habituation period, the test was started by changing the air flux so that it flowed from the goal to the start and opening the guillotine door. The trial was terminated after 1 min for the first three trials and after 5 min for the fourth trial, by the subject entering the goal area. The mean intertrial interval was approximately 80 min.

Run time, as defined as the elapsed time from the subject leaving the start box to entering a goal area, was measured by off-line analysis with video recording. Shorter times may reflect greater motivation to approach receptive females in the stimulus box [29]. In the fourth trial, the duration of time spent in the goal area was measured, in which longer durations may also reflect greater motivation toward receptive females. All values are presented as the percent of the total test time spent in the goal area.

### Sampling of brain tissue and blood following stimulation

One week after the runway test (when the animals were approximately 14 weeks old), one third of males were exposed to receptive females, and brain tissue and blood samples were collected for analysis. The apparatus was an acrylic box was divided into two compartments (30×30×25 cm, each) by a doubled wire-mesh wall. All of the males were previously exposed to the apparatus during two 10-min sessions. On the sampling day, each male was placed in one compartment of the box with a receptive female (same partner of copulatory test) in the next compartment. The male subject was maintained in the compartment with the female for 20 min, because previous studies have reported in 5-HT, DA, and hormonal responses within that time window by, food intake [30], restraint stress [7], [31], or presentation of a receptive female [32]. After 20 min of exposure, the male subject was euthanized in an adjacent room by rapid decapitation. Another third of the males were subjected to the same manipulation and sacrificed, but they were exposed to an empty compartment rather than a female exposure. The remaining third of males were immediately euthanized after removal from their home cages. The final numbers of animals in each group was as follows (n = 14, in each rearing group): female exposure (Female), n = 5; no female exposure (No Female), n = 5; and euthanized in the home cage (Home), n = 4.

The brains of these subjects were removed from the skull and roughly dissected into tissue blocks including the regions of interest. The tissue blocks were dipped in dry-iced isopentane and kept in −80°C until assay.

The tissue blocks were coronally sectioned at 500 µm thickness by a freezing microtome. The sections including the regions of interest were put on an ice-cold dissection plate, and bilateral punches were taken from the nucleus accumbens (NAC), striatum (STR), and preoptic area (POA) according to a brain atlas [33]. Collected tissue samples were transferred into a micro tube, and the weight was measured (2–7 mg, each area) in preparation for high-performance liquid chromatography (HPLC) analysis. Tissue samples were homogenized with 100 µl of PCA solution (0.2 M HClO_4_, 100 µM EDTA-2Na), and centrifuged at 20,000×g for 15 min at 0°C. Aliquots of supernatant (70 µl) were adjusted to a pH 3.0 and diluted with mix solution (2 mM CH_3_COOH, 10 µM EDTA-2Na). The prepared solutions were frozen and kept at −80°C until the assay was performed.

The subject's blood was also collected in a plastic tube (4 ml, on the ice) during the decapitation and centrifuged at 5,000×*g* for 10 min at 4°C. Supernatant (1 ml, plasma sample) were frozen at −80°C until the assay was performed.

### Assay for 5HT and DA

To assess the levels of 5-HT or DA in various brain regions, we adopted a HPLC method using homogenized samples. 5-HT or DA levels in the tissue samples were quantified by a HPLC system (EP-300, Eicom, Kyoto, Japan), as described previously [31]. The prepared solutions from the samples were injected into a pre-column (PC-03AC, Eicom) with a mobile phase consisting of 0.1 M phosphate buffer at pH 6.0, 0.13 mM EDTA, 2.3 mM sodium-1-octanesulfonte and 20% methanol. 5HT and DA were then separated with a separation column (CS-5ODS, Eicom) and detected with an electrochemical detector (ECD-300, Eicom). The separation column was maintained at 25°C, and the electrode potential was set to 400 mV against a reference electrode. The retention times for 5HT and DA were identified from the respective peaks of external standards. Each area under the chromatographic peaks was determined with an integrator (PowerChrom v2.1.4J, ADInstruments, Tokyo, Japan) to analyze the detected amounts of each compound. All data were compared with the mean basal levels in the Home group, which were set to 100%.

### Assay for corticosterone and testosterone

Plasma corticosterone and testosterone levels were measured by an enzyme immunoassay. The method described earlier [34] was modified. Steroid hormones were extracted from 50 µl plasma with ether. Each well (ELISA Plates 9018, Corning, NY, USA) was coated with secondary antibody solution (anti-rabbit γ-globulin serum, Seikagaku Co., Tokyo, Japan). Standard corticosterone (Wako) and testosterone (Sigma) were diluted in the assay buffer (phosphate buffered saline [pH 7.4] with bovine serum albumin 1.0 g/l). For an enzyme immunoassay of corticosterone and testosterone, 25 µl of standard and sample solution, 100 µl of anti-serum solution (anti-corticosterone: FKA420-E, COSMO Bio, Tokyo, Japan and anti-testosterone: FKA102-E, COSMO Bio) and 100 µl of horse-radish-peroxidase (HRP) labeled steroid hormones (corticosterone-3-CMO-HRP, FKA419, COSMO Bio and testosterone-3-CMO-HRP, FKA101, COSMO Bio) were pipetted into each well. The plates were incubated overnight at 4°C. Non-bound ligands were removed and 150 µl of substrate solution for HRP was added to each well and incubated for 40 min at room temperature. The reaction was stopped by the addition of 50 µl H_2_SO_4_ (4N). The absorbance at 450 nm wavelength was recorded by a microplate reader (model 550, Bio-Rad, CA, USA), and the contents of steroid hormones were calculated using the software (Microplate Manager, Bio-Rad). The minimum detectable level of corticosterone and testosterone were 9.9 and 4.9 pg/well. All intra- and inter-assay coefficients of variation were less than 15%.

### Statistical analysis

All values are reported as the mean ± the standard error of the mean (SEM). Behavior in the open-field test and time taken to reach the goal area in the runway test were analyzed with Student's t-tests. An analysis of variance (ANOVA) for repeated measures with a factor of rearing condition (i.e., SE versus EE) × a factor of time (i.e., number of sessions) was performed to analyze the sexual behavior and time spent in the runway test data. A two-way ANOVA with the main factors of rearing condition and the stimulation (i.e., female, no female, or home) was performed to analyze the 5-HT, DA, corticosterone, and testosterone data. When appropriate, ANOVAs were followed by Bonferroni post-hoc tests. All statistical analyzes were performed with the software package SPSS (v 16.0 J). Differences were considered statistically significant at *P*<0.05.

## Results

### Copulatory behavior

**Figure 1:**
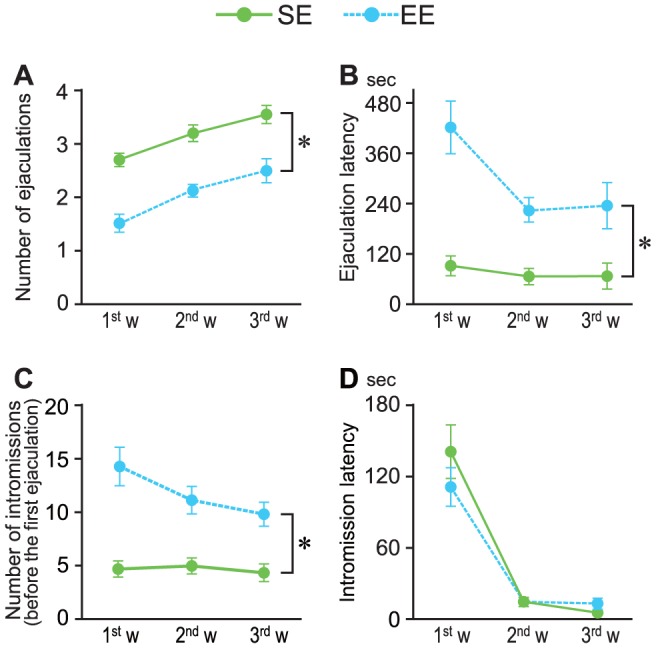
Effects of rearing condition on male copulatory behavior in 3 consecutive weekly sessions. Male rats reared in an enriched environment (EE, blue dots) showed a decreased number of ejaculations (A) and intromissions (C) and prolonged ejaculation latencies (B) compared with rats reared in a standard environment (SE, green dots). On the other hand, there were no group differences in intromission latencies (D). *P<0.001 compared with SE, ANOVA for repeated measures.

To evaluate the effects of rearing conditioning on sociosexual activity, copulatory behavior was tested for 3 consecutive weeks in a circular open field. EE rats showed decreased ejaculatory behavior based on a decreased number of ejaculations (F_1, 26_ = 41.68, *P*<0.001, Figure 1A) in concert with prolonged latencies (F_1, 26_ = 28.14, *P*<0.001, Figure 1B). The other parameters measured also indicated decreased sexual activity in EE rats, including the PEI (F_1, 26_ = 21.45, *P*<0.001, Table 1), MF (F_1, 26_ = 7.361, *P*<0.05, Table 1), number of intromissions (F_1, 26_ = 19.34, *P*<0.001, Figure 1C) and III (F_1, 26_ = 14.26, *P*<0.001, Table 1). On the other hand, there were no differences between the groups in their intromission latency or IR (Figure 1D and Table 1).

**Table 1:** Male sexual behavior of SE and EE rat.

	SE			EE		
	1^st^ w	2^nd^ w	3^rd^ w	1^st^ w	2^nd^ w	3^rd^ w
PEI (sec)	224.4±7.0	207.6±7.4	194.6±9.2	282.5±12.8	250.6±7.9	236.9 8.5**
MF	4.0±1.0	7.9±1.3	7.0±1.3	15.1±3.4	7.1±1.1	14.1±3.5*
III (sec)	19.4±2.2	11.9±1.6	11.6±2.0	28.8±2.6	19.7±1.6	22.1±3.9**
IR	0.79±0.04	0.69±0.03	0.71±0.03	0.62±0.05	0.75±0.03	0.63±0.06

Data are mean ± SEM. PEI, post ejaculated interval; MF, mount frequency; III, inter intromission interval; IR, intromission ratio. *P<0.05, **P<0.01 compared with SE group, ANOVA for repeated measures.

### Open-field behavior

**Figure 2:**
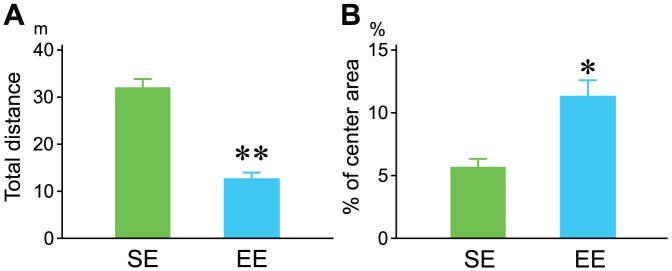
Behavioral emotionality of male rats reared in an EE (blue bar) in an open field that was the same field used in the copulatory behavior test. Group differences were observed in total distance travelled (A) and the proportion of time spent in the center area (B). ** *P*<0.001, *P<0.005 compared with SE (green bar), Student's t-test.

Because EE markedly affected copulatory behavior, basal emotionality was analyzed in the same open field. EE rats showed less locomotion than SE rats in an open field (*P*<0.001, Figure 2A). Furthermore, EE rats spent a greater proportion of their time in the center of the field (*P*<0.005, Figure 2B).

### Male behavior toward a female in the runway test

**Figure 3:**
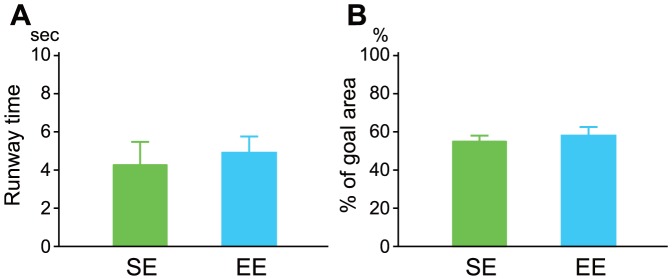
Male motivational behavior toward a female in a runway test. Male subjects in both groups ran faster through a long alley (150 cm) toward a goal box containing an estrus female. Note that male rats in both group (EE, blue bar; SE, green bar) showed comparative motivation to reach the females in terms of their runway time (A) or percent time spent in the goal area (B).

To evaluate the male sexual-motivation toward a receptive female, we performed the runway test. In both environmental groups, the mean run time of the males to the goal area was faster when there was an estrous female than when there was not (F_3, 20_ = 18.58, *P*<0.001, SE: 37.6±8.28, time to empty goal box and 4.2±1.29, mean time to goal box containing an estrus female, EE: 18.5±6.69 and 4.9±0.84). However, there were no significant differences between the groups in their runway time to a female (Figure 3A). In addition, after reaching the box, male rats in both spent considerable time in the narrow goal area adjacent to the estrus female (Figure 3B).

### Effects of rearing on 5-HT and DA activity following female exposure

**Figure 4:**
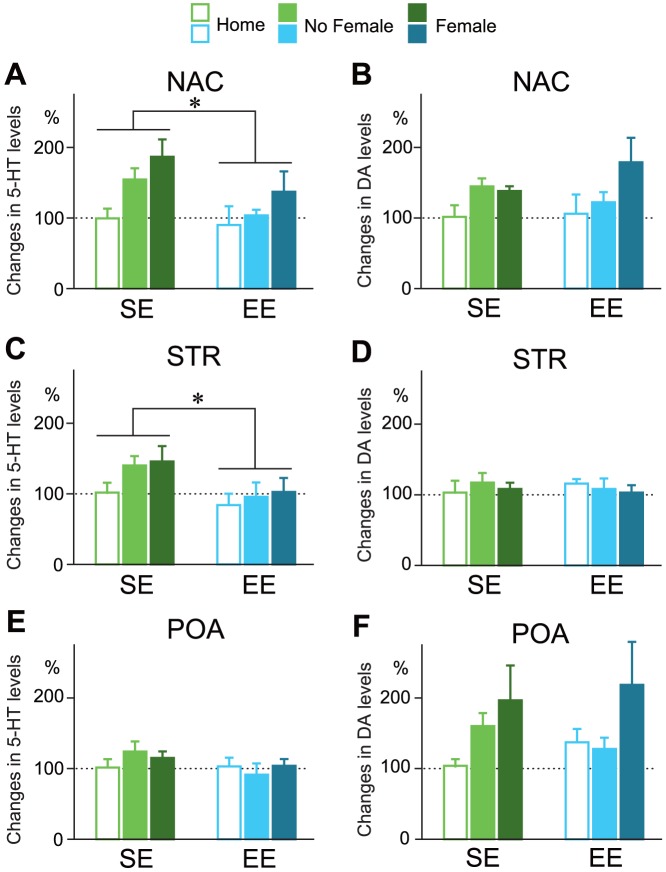
Effects of rearing condition on neuroendocrine responsiveness and serotonin (5-HT; A, C and E) and dopamine (DA; B, D and F) levels following stimulus exposure (Home, white bar; No Female, light-color bar; Female, dark-color bar). EE rats (blue color) showed decreased 5-HT responsiveness in the nucleus accumbens (NAC, A) and striatum (STR, C), but not in the preoptic area (POA, E) compared with SE rats (green color). Main effects for stimulus condition were observed for both 5-HT and DA in the NAC (A and B). *P<0.05 compared with SE, two-way ANOVA (factor rearing-conditioning-group × factor stimuli).

Male rats were exposed to an experimental cage with or without a female to evaluate changes in catecholamine levels in the central nervous system. For 5-HT, two-way ANOVA revealed a significant increase by female exposure, with a main effect stimuli exposure in the NAC (F_2, 22_ = 5.027, *P*<0.05, Figure 4A). Moreover, a main effect of group revealed that EE rats showed a suppressed 5-HT response following female exposure in the NAC (F_1, 22_ = 4.57, *P*<0.05, Figure 4A) and STR (F_1, 22_ = 5.47, *P*<0.05, Figure 4C). No significant changes in 5-HT levels were found in the POA (Figure 4E). A post-hoc test showed that there was a tendency towards an overall decreased in individual responses in EE rats in the No Female (NAC; *P* = 0.089, STR; *P* = 0.090) and Female conditions (NAC; *P* = 0.091, STR; *P* = 0.090) compared to the SE rats. The statistical analysis indicated no consistent differences in basal levels of 5-HT content between the groups. For DA, two-way ANOVA indicated only a main effect of female exposure in the NAC (F_2, 22_ = 3.49, *P*<0.05, Figure 4B) that did not significantly differ by rearing group (Figure 4B, D, F), and a tendency towards a response to female exposure in the POA (F_2, 22_ = 3.02, *P* = 0.070, Figure 4F). The statistical analysis indicated no consistent differences in basal levels of DA content between the groups.

### Effect of rearing conditioning on corticosterone and testosterone following female exposure

For corticosterone, two-way ANOVA revealed a significantly suppressed response in EE rats following female exposure, with a significant main effect group (F_1, 22_ = 7.63, *P*<0.05, Table 2) but not for exposure condition. A post-hoc test showed significant differences between the groups, with a decreased corticosterone response to female exposure (Female situation) in EE rats (*P*<0.05). For testosterone, two-way ANOVA revealed a significantly suppressed response in EE rats following female exposure, with significant main effects for group (F_1, 22_ = 4.56, *P*<0.05, Table 3) and exposure condition (F_2, 22_ = 6.315, *P*<0.01, Table 3). A post-hoc test determined that female exposure increased testosterone levels compared to the Home condition in the SE group (*P*<0.05) but not in EE group. Testosterone contents during the Home condition and in response to stimuli (No Female and Female situation) did not significantly differ between the groups.

**Table 2:** Effect of rearing environment on plasma corticosterone level following the exposure to stimuli.

	Corticosterone (ng/ml)	
stimuli	SE	EE
Home	266±16	186±24
No Female	305±38	244±48
Female	301±61	170±17*

Data are mean ± SEM. Two-way ANOVA revealed a significantly suppressed response in EE rats following female exposure with a main effect group. *P<0.05 compared with SE, post-hoc test.

**Table 3:** Effect of rearing environment on plasma testosterone level following the exposure to stimuli.

	Testosterone (ng/ml)	
stimuli	SE	EE
Home	3.56±0.40	2.28±0.34
No Female	5.06±0.95	2.97±0.74
Female	8.19±1.8*	5.54±1.3

Data are mean ± SEM. Two-way ANOVA revealed a significantly suppressed response in EE rats following female exposure with main effects for group and stimuli exposure.*P<0.05 compared with Home stimuli, post-hoc test.

## Discussion

In the present study, rearing in an EE led to notably decreased copulatory behavior, a lower number of ejaculations, prolonged ejaculation latencies, longer postejaculatory intervals and other changes (Figure 1 and Table 1). There have only been a few reports of the effects of EE rearing on male copulatory activity. In agreement with the present results, Swanson et al. [35], [36] reported that males reared in an EE showed prolonged latencies to ejaculation compared with control males, and only a few EE males managed to achieve an ejaculation in their study.

To date, there is no direct evidence for the neurobiological regulation of changes in copulatory behavior in EE male rats. Although several possible explanations could account for our present results, we hypothesize that the decreased copulatory behavior in EE rats is related to lower emotional responsiveness and central regulation of 5-HT and hormonal responses during mating. First, the nature of our behavioral assessments may explain the emotional responsiveness to copulatory activity. The present behavioral results indicated that EE males decreased their emotional responsiveness in the same open field used in the sexual behavior test (Figure 2). In a previous report, we showed that EE decreases emotional responsiveness and that this is related to alteration of specific interneurons in the basolateral amygdala [11]. Interestingly, EE rats increased the percentage of locomotion exhibited in a center arena and lowered locomotor activity in an open field over a similar observational window as used in the present study, whereas EE rats traversed more quickly across an elevated narrow beam in an anxiogenic situation [11]. These results suggest that EE leads to lower emotional responsiveness, and that notion has been supported a number of studies related to emotional behavior of EE males, i.e., decreased locomotion in an open field [37], [38], [39], enhanced activity in an elevated plus maze [8], [40], [41] and increased novelty seeking in a hole board test [42], [43]. During mating, male rats typically show emotional responses to an estrus female. In view of the emotional responsiveness aspects of sexual activity, it has been reported that painful stimuli, such as electrical foot shocks or tail pinches, facilitate male rat sexual behavior [44], whereas anxiolytic drugs increase the number of mounts preceding ejaculation and prolong ejaculation latencies [45]. Taking into consideration the effects of emotional responsiveness on sexual activity, these data suggest that rearing in an EE suppresses superfluous emotional responsiveness to an unfamiliar place and presumably neophobic traits, resulting in a lower level of sexual activity.

Second, corticosterone and testosterone responsiveness in the present study may support our hypothesis of lower emotional responsiveness of EE rats in mating situations. Following the non-physical contact exposure to an estrus female, SE males showed clearly elevated levels of plasma corticosterone and testosterone. However, EE rats exhibited significantly suppressed hormonal response (Table 2 and 3) in accord with our hypothesis of distinct emotional responsiveness. Given that the basal levels (Home situation) were the same between the groups, differential hormonal responsiveness may be responsible for the different reaction across the groups following female exposure. Morley-Fletcher et al. [46] reported that prenatal stress increases the corticosterone secretion in response to restraint stress, but this increase is absent in EE rats. Bonilla-Jaime et al. [27] reported that following non-physical contact exposure to an estrus female, but not to a non-receptive female, only sexually experienced males showed increased levels of plasma corticosterone and testosterone. These data suggest that increased responses of plasma corticosterone and testosterone reflect, at least in part, emotional responsiveness in the face of an estrus female.

Third, the differential serotonergic responses we observed may indicate a possible role for neuroendocrine regulation in the emotional responsiveness and lower copulatory activities of EE rats. Serotonergic systems play an important role in the regulation of behavioral, autonomic, and endocrine responses to some types of stress. Exposure of animals to various acute stressors, such as restraint, electroshock, tail pinch, and forced swimming stress, has been shown to increase 5-HT release in the prefrontal cortex, STR, NAC, amygdala, hippocampus, and lateral hypothalamus [47], [48]. Because such brain areas are all involved in emotional behavior, we evaluated the catecholamine content from various areas simultaneously using homogenized samples. In the present study, the increase in 5-HT levels in the NAC and STR following female exposure was significantly attenuated in the EE group (Figure 4). These data indicate that SE males show excessive emotional responsiveness and neurochemical responses to female exposure, whereas EE males present comparable responsiveness to when they are in their home cage (Home situation). In particular, 5-HT is one of the most plausible candidates as a regulatory factor for male copulatory behavior [24], [49], [50]. Drugs that enhance central serotoninergic synaptic activity are effective in improving the control of ejaculation in patients with premature ejaculation.

Our observed dopaminergic responses may provide further implications for male emotional responsiveness to estrus females. In the present study, dopaminergic responses did not differ between the rearing groups (Figure 3), and both groups of rats showed increased DA content in the NAC following female exposure (Figure 4). Many authors have discussed the importance of DA systems for male sexual motivation [23], [51], [52]. From this point of view, our data for the runway test suggest that sexual motivation in EE rats is comparable to that in SE rats, that is, EE rats ran to a goal box containing an estrous female just as fast as SE rats. In addition, male rats in both groups showed almost the same intromission latencies in copulatory behavior in accord with the results of the runway test. Taken together, decreased copulatory activities in EE males may result from neither lower sexual motivation nor lower DA system activity as a copulatory regulating factor.

Finally, the present results lead us to focus on the 5-HT system in the NAC and STR, which are innervated by serotonergic neurons from the medial and dorsal raphe nuclei, as the primary neurobiological mechanism for the differences in copulatory activity between EE and SE males. Previous studies have reported plasticity of the 5-HT system via interventional EE [39], [53], [54]. In addition, wheel running, which is one of most substantial energetic activities in which an EE rat will participate, has been shown to modulate central 5-HT neurotransmission [55], [56]. The NAC has been proposed to be a key region for emotional (anxiety-like) behavior and sexual activity [57], [58], [59], [60], [61]. Taken together, decreased sexual activity may be influenced by 5-HT regulation in the NAC.

In conclusion, rearing in an EE decreases copulatory activity, which is associated with lower emotional responsiveness and modulation of 5-HT neuronal activity. In the light of recent studies and social interest in the effects of early life experiences on postpubertal behavior, our findings provide important new insights into the role of the 5-HT system in the emotional consequences of rearing history on male sociosexual activity.
